# Preclinical evidence supporting the clinical development of central pattern generator-modulating therapies for chronic spinal cord-injured patients

**DOI:** 10.3389/fnhum.2014.00272

**Published:** 2014-05-30

**Authors:** Pierre A. Guertin

**Affiliations:** ^1^Department of Psychiatry and Neurosciences, Laval UniversityQuebec City, QC, Canada; ^2^Spinal Cord Injury and Functional Recovery Laboratory, Laval University Medical Center (CHU de Quebec)Quebec City, QC, Canada

**Keywords:** CPG, locomotion, SGE, ejaculation, LDC, defecation, SMC, micturition

## Abstract

Ambulation or walking is one of the main gaits of locomotion. In terrestrial animals, it may be defined as a series of rhythmic and bilaterally coordinated movement of the limbs which creates a forward movement of the body. This applies regardless of the number of limbs—from arthropods with six or more limbs to bipedal primates. These fundamental similarities among species may explain why comparable neural systems and cellular properties have been found, thus far, to control in similar ways locomotor rhythm generation in most animal models. The aim of this article is to provide a comprehensive review of the known structural and functional features associated with central nervous system (CNS) networks that are involved in the control of ambulation and other stereotyped motor patterns—specifically Central Pattern Generators (CPGs) that produce basic rhythmic patterned outputs for locomotion, micturition, ejaculation, and defecation. Although there is compelling evidence of their existence in humans, CPGs have been most studied in reduced models including *in vitro* isolated preparations, genetically-engineered mice and spinal cord-transected animals. Compared with other structures of the CNS, the spinal cord is generally considered as being well-preserved phylogenetically. As such, most animal models of spinal cord-injured (SCI) should be considered as valuable tools for the development of novel pharmacological strategies aimed at modulating spinal activity and restoring corresponding functions in chronic SCI patients.

## Introduction

Locomotion is the act of self-propulsion by an animal (Hugues and Wiersma, 1960; Delcomyn, 1977; Kandel et al., 2000; Hopper and DiCaprio, 2004). Forms of terrestrial locomotion generally include walking, running, and hopping. In vertebrates, its control depends upon several neural systems that ensure propulsion, body orientation (equilibrium or postural control), and steering (goal-direction control) (Ivanenko et al., 2006). Certain areas of the brain have for role, through signals sent via descending neural pathways to the spinal cord, to trigger and modulate basic locomotor outputs generated spinally. The latter are organized essentially by a network localized in the lumbar segments of the spinal cord, generally referred to as the Central Pattern Generator (CPG) for locomotion. That network is responsible for much of the timing and pattern of the complex, rhythmic, coordinated muscle activities that underlie locomotion. In addition to this direct contribution from central systems, there are also different feedback and feedforward loops that use peripheral cues for proper adaptation of gait under different circumstances. Since most methods and experimental tools needed for studying these systems at the cellular level are rather invasive, it has remained difficult to study them in great details in humans. However, research in different animal models has revealed significant details about the organization and function of each constitutive element of these systems (Buschges et al., 2008). In most cases, great similarities have been found between neuronal circuits that generate rhythmic motor patterns among species. In fact, just examining similarities between various forms of locomotion among species already provide preliminary evidence of well-preserved spinal mechanisms throughout evolution. The neural system controlling locomotion is probably the most studied systems thus far among all spinal systems underlying comparable stereotyped motor behaviors such as micturition, ejaculation or defecation (Guertin and Steuer, 2009). Nonetheless, from compiling data gathered about these other systems more recently, it may be said that great similarities seem to exist among spinal systems that control these fundamental behaviors.

## Different forms of locomotion among vertebrate species

Although some differences exist between forward and backward locomotion in cats, mice humans, salamanders or lampreys, to name a few, most of the basic alternating patterns between flexor-like and extensor-like muscles are generally maintained even in invertebrate species (Székely et al., 1969; Pearson and Duysens, 1976; Kristan and Weeks, 1983; Robertson and Pearson, 1985; Pearson, 1993; Currie and Lee, 1997; Cheng et al., 1998; Currie, 1999; Matsumoto et al., 2007; Liu et al., 2012). For instance, lampreys and salamanders swim by contracting muscles on either side of the body in order to generate waves of flexion that travel the length of the body from nose to tail (rostrocaudal propagation of undulations), generally getting larger as they go along (Getting, 1977). Locomotion on land raises different complications, such as the effects of gravity. Nonetheless, walking—the most common gait in legged animals, shares comparable key features with swimming. Indeed, it is essentially composed of a series of rhythmic contractions between antagonist muscles of the main moving segments, the legs instead of the whole body. More specifically, it is the result of successive coordinated contractions between flexors and extensors of all limbs accompanied of bilateral alternation for bipeds and quadrupeds.

## Several neural networks involved

As mentioned earlier, it is now generally accepted that the basic motor patterns and rhythms underlying walking (i.e., the most extensively studied form of locomotion) are organized and generated in the spinal cord by the CPG (Grillner, 1981; Guertin, 2009a). However, many other networks in the CNS have key roles to play for succesfull locomotion to be achieved. For instance, supraspinal centers in the brainstem and forebrain are essential for initiating and controlling locomotor movements. One of them located in the cerebellum plays an important in sensorimotor control and in intra- and inter-limb coordination. The vermis and the intermediate region of the cerebellum receive information through the spinocerebellar pathways about the ongoing activities in the CPG and the somatosensory receptors. The information is conveyed to Purkinje neurons, transformed, returned to brainstem descending tract neurons also involved in the initiation of locomotion. Another important center is the mesencephalic locomotor region (MLR), first discovered in cats and later found in all vertebrate species tested to date (Orlovskii et al., 1966; Orlovski et al., 1966; Orlovsky and Shik, 1976; Jordan et al., 2008; Juvin et al., 2012). The reticulospinal tract that originates in the reticular formation has also been found to be essential for the production of locomotor activity evoked by brainstem stimulation in many different species, its activation is necessary for the initiation of locomotion in normal conditions (Grillner, 1981; Cheng and Magnuson, 2011; Le Ray et al., 2011). Along this idea, locomotor-initiating centers in the midbrain/pontine tegmentum and cerebellum converge in the reticular nucleus before descending to the spinal cord.

## Spinal cord organization

It is impossible to provide an update on neural systems that control locomotion or other stereotyped motor behaviors, specifically those in the spinal cord, without reviewing first, fundamental details about spinal cord organization (Guertin, 2012). The spinal cord has often been considered as a simple relay between brain cells and effective organs (muscles, skin, etc.). However, it is increasingly recognized that the spinal cord is also a “command center” involved in the control and modulation of several functions (Kandel et al., 2000). With its one billion neurons (Kalat, 1998), it is definitely a significant structure of the CNS that can control both simple motor acts such as reflexes (e.g., monosynaptic excitatory, reciprocal inhibitory, withdrawal and crossed-extension reflexes) as well as more complex motor functions such as locomotion, bladder/bowel control, and sexual function (Guertin, 2012).

## Gross anatomy of the spinal cord

The spinal cord constitutes the most caudally located structure of the CNS (Netter, 2006). Contained within the vertebral column, it extends from the medulla to the first lumbar vertebra (Patestas and Gartner, 2006). In humans, its long and thin elliptical structure varies in length between 43 and 45 cm comprising 31 segments—8 cervical, 12 thoracic, 5 lumbar, 5 sacral, and 1 coccygeal. Every segment is associated with a pair of nerves (and roots) on each side that comprises sensory nerve roots entering the spinal cord at each level, and motor roots emerging from the cord at each level. In rostral parts, the spinal nerves exit directly from (just above from C1 to C7 or just below from C8 and lower) the vertebra associated numerically with the corresponding spinal cord segment. However, in caudal parts of the spinal cord, the spinal nerves travel further down the column before exiting (also known as the cauda equine). In transverse sections, the spinal cord displays white and gray matter tissues. The white matter, located more peripherally, contains white matter tracts, ascending and descending myelinated fibers, carrying both sensory and motor inputs. The gray matter, more centrally located, is characterized by its butterfly-shape that contains unmyelinated cells and, specifically, simple as well as more complex spinal circuits. In the center, there is the central canal that contains cerebrospinal fluids formed mainly in the ventricular system (choroid plexus). Peripherally, the meninges are layers of tissue surrounding the spinal cord for its protection—the dura, the arachnoid and the pia. The latter is relatively thin and tightly associated with the surface of the spinal cord. The spinal cord is well-vascularized with blood vessels including the anterior spinal artery, the bilateral sulcal branches, the bilateral posterior spinal arteries, the pial arterial plexus as well as the anterior/posterior spinal veins, the anterior/posterior sulcal veins, and the pial venous plexus.

## Spinal Cord White Matter

Several tracts carry information between supraspinal (MLR, reticular formation, etc.) and spinal structures (e.g., motoneurons, simple reflexes, CPG, etc.). Ascending tracts convey sensory signals associated with the sense of touch, pressure, proprioception, and vibration via relatively large myelinated fibers (e.g., gracile fasciculus, cuneate fusciculus). They travel contralaterally (decussation) in the medulla prior to be redirected toward the thalamus and the sensory cortex. In contrast, the lateral or so-called anterolateral columns convey information about pain and thermal sensation and decussate instead at the spinal cord level (close primary afferent entry) prior to ascend to the brain via a number of tracts (e.g., Lissauer’s tract, dorsospinal tract, ventrospinal tract, spinocerebellar tract, spinothalamic tract, etc.).

The descending motor system is also divided in multiple tracts essentially composing the so-called pyramidal and extrapyramidal tracts. The corticospinal tracts, also called the pyramidal tracts, contain about one million axons on each side involved mainly in skilled movements. The extrapyramidal tracts are involved instead in the control of posture and locomotion (Bretzner and Drew, 2005; Barthélemy et al., 2011). The pyramidal tract originates from the cerebral cortex and from some brainstem motor nuclei. It constitutes the most direct descending motor pathway (e.g., some monosynaptic connections) between the motor cortex (Brodmann’s areas 1, 2, 3, 4, and 6) and the final common motor pathway, namely the spinal motoneurons all segmental levels. About 80–90% of these corticospinal axons decussate contralaterally at the pyramid level in the medulla oblongata. From there, they form the lateral corticospinal tract that sends input to spinal motoneurons in the ventral horn. 10–20% descends instead ipsilaterally as the ventral corticospinal tract and decussates in the spinal cord prior to synapsing with spinal motoneurons. A few others form instead the corticobulbar tract that sends input to brainstem motoneurons for face, head and neck muscle control. Extrapyramidal tract neurons (rubrospinal, vestibulospinal, tectospinal and reticulospinal) originate from subcortical nuclei in the pons (reticular formation) and medulla oblongata where they send projections to spinal motoneurons of all segments. The rubrospinal tract receives inputs from the motor and premotor areas 4 and 6 prior to send projections contralaterally (decussation in the brain) to spinal motoneurons cervically. It is generally considered to facilitate flexion and inhibit extension in the upper extremities. The tectospinal tract sends axons in premotor lamina of the spinal cord (VI-VIII) cervically for neck and head motor control whereas the reticulospinal track sends projections to all levels of the spinal cord for autonomic control (e.g., cardiovascular and respiratory functions, blood pressure, micturition, defecation). The vestibulospinal tract sends projections laterally or ventrally on the ipsilateral side of the spinal cord to laminae VII and VIII for extensor control (English, 1985).

## Simple Reflex Pathways of the Gray Matter

As described above, white matter tracts are mainly “relays” sending signals up (sensory-related) and down (motor control) between supraspinal and spinal structures. In contrast, gray matter neurons in the spinal cord form reflex pathways and complex neuronal networks. Simple reflex arcs or pathways are normally associated with well-known classical reflexes (Matthews, 1972, 1991). By definition, a simple reflex, in normal condition, typically leads to a rapid, predictable, repeatable, stereotyped and involuntary motor reaction in response to the corresponding specific stimulus. Most reflexes are mediated locally, within one or two spinal segments, by relatively simple neural pathways in the spinal cord—involving generally either one (monosynaptic), two (disynaptic) or more (polysynaptic) synapses and corresponding neurons (i.e., interneurons and motoneurons). Simple reflexes may be of autonomic (related with inner organs, eyes, blood vessels, etc.) or somatic (related with skeletal muscle responses) origin. The latter has been more extensively studied mainly in the spinal cord-transected or decebrate cat models. Among the main somatic spinal reflexes, the Ia, Ib, II, and FRA (flexion reflex afferent) reflexes have been particularly well-characterized although their roles in the control of locomotion or other stereotyped motor behaviors remain incompletely understood (Henneman, 1974; Forssberg and Hirschfeld, 1988; Field-Fote et al., 2012). This said, activation of some of these reflexes via electrical stimulation of muscles, nerves or lumbar spinal cord area in spinal cord-injured (SCI) patients was shown to temporarely elicit or promote some waking movements with or without pharmacological aids (see recent work from Edgerton’s or Gerasimenko’s groups) (Edgerton et al., 2001).

### Monosynaptic Reflex

The so-called Ia monosynaptic reflex arc is a well-characterized somatic spinal reflex pathway. It is considered the simplest and fastest reflex of all, mediating primary afferent (Ia) inputs from muscle spindles typically activated by muscle stretch (e.g., following a tendon jerk, tendon tap or myotatic reflex). Ia inputs establish monosynaptic connections with homonymous alpha-motoneurons in the ventral horn gray matter. This excitatory reflex increases homonymous muscle contraction in response to muscle elongation while inhibiting antagonist muscle contraction via collateral branching. It is generally considered to play a role in tonus and postural adjustments which is important for terrestrial locomotion. Its level of activity is phase and task-dependent—i.e., larger response during standing compared with walking or running as well as during extension (stance phase) compared with flexion (swing phase) (Stein and Capaday, 1988). Findings in decerebrate and paralyzed cats provided clear evidence of its role also in reflexively-increased extensor activity during stance and in resetting rhythm or cycle probably via direct input upon CPG elements (Guertin et al., 1995; Angel et al., 1996, 2005). Hence, Ia inputs during locomotion may serve to compensate for an unsuspected increase of loading during ambulation as well as to increase tonus in extensor muscles during stance (Guertin et al., 1995; Pearson, 1995; Guertin, 1996).

### Ib Reflex Pathways

Also known as the inverse myotatic reflex or autogenic inhibitory reflex pathway, the Ib reflex arc is associated with peripheral afferent inputs from Ib afferent fibers and Golgi tendon organs. It has been shown to inhibit homonymous and synergistic alpha-motoneurons at rest via a disynaptic arc (two synapses involving one inhibitory neuron called the Ib interneuron), originally believed to serve as a protective mechanism against excessive muscle contraction. This view has eventually changed when discovering that a wide range of muscle activity and load can alter Ib firing. It is also task-dependent since during locomotion, Ib inhibition is replaced by Ib excitation to homonymous and synergistic alpha-motoneurons at all joints of the lower extremities via a rhythmically active candidate excitatory interneuron located in the lumbar enlargement area (lamina VII) (Gossard et al., 1994; Angel et al., 1996, 2005). Its role during locomotion may be also to enhance muscular contraction of extensors during the stance phase and to reset stepping to extension when activated during the swing phase (Guertin et al., 1995; Pearson, 1995).

### Flexion (withdrawal) Reflex Pathways

The FRA pathway is activated specifically by high-threshold afferent fibers (e.g., associated with cutaneous nociceptor A or C fibers, group II, III and IV muscle afferent fibers, etc.) (Knikou et al., 2009). Ipsilaterally, it involves at least two interneurons (three or more synapses) over several segments prior to synapse upon alpha-motoneurons ipsilaterally for extended flexor contraction and extended extensor inhibition. With weaker stimulus, only ipsilateral effects are found. However, with stronger stimulus, opposite contralateral effects may also be found (excitation of extensor muscles and inhibition of flexors)—the crossed-extension reflex. These reflex pathways play a role in reflex withdrawal of a limb (unilateral flexion) from a painful stimulus. Task-dependency has also been found since, during locomotion, a long-lasting burst of activity is unraveled ipsilaterally during pharmacologically-induced fictive locomotion (long-lasting FRA response) probably via CPG elements (specifically the flexor portion of the CPG) since FRA stimulation under experimental conditions was shown to reset the step cycle to flexion (Jankowska et al., 1967a,b; Perreault et al., 1995; Schomburg et al., 1998; Ollivier-Lanvin et al., 2011). Clinically, it is associated with the Babinski sign (i.e., tongue depressor-induced plantar extension) as an indication of neurological problems in adults (e.g., spinal pyramidal tracts-induced injury caused by trauma or tumor). The long-lasting FRA response as well as myoclonus can also be uncovered following FRA stimulation in patients with SCI.

## Central Pattern Generators—Early Evidence and Underlying Concepts

To date, the best-characterized spinal network is undoubtedly the CPG for locomotion that directly controls the basic motor commands underlying ambulation (Guertin, 2009a; Guertin and Steuer, 2009). Seminal work from Flourens, Phillipson, Sherrington, and Graham Brown supported by subsequent evidence generated largely from the 1960s onwards showed that, across species, rhythmic and stereotyped motor behaviors including walking, flying, and swimming are controlled largely by a neuronal network generally referred to as the CPG for locomotion (Flourens, 1824; Freusberg, 1874; Sherrington, 1910; Graham Brown, 1911, 1914; Lhermitte, 1919; Grillner, 2006; Clarac and Pearlstein, 2007). Early observations from paraplegic dogs revealed the existence of locomotor-like movements that can occur spontaneously after a complete transection (TX) of the spinal cord. That was elicited specifically when dropping one of the limbs from a flexed position. Comparable observations by Philippson led him to conclude that the spinal cord could control locomotion using both central and reflex mechanisms. Sir Charles Sherrington’s work in TX cats and dogs provided additional evidence that such spinal locomotor-like movements were the result of reflex actions from proprioceptors onto some spinal centers (Sherrington, 1910). However, it is Thomas Graham Brown, who described more directly the existence of a spinal neuronal network as main command center for locomotion (see also Stuart and Hultborn, 2008 for a thorough description of Sherrington and Graham Brown’s original contributions) in anesthetized animals lying on one side when stepping movements in the hindlimbs were spontaneously expressed (“narcosis progression”) after TX thoracically (Graham Brown, 1911, 1914; Stuart and Hultborn, 2008). More evidence of its existence cellularly (half-center now generally referred to as the CPG) occurred when intracellular recordings became possible in the 1960s. A Swedish group led by Anders Lundberg recorded interneurons located in the lumbar segments of the spinal cord (lamina VII), active following FRA stimulation. Since then, several theories have been proposed to tentatively describe the functional organization of that CPG—the Miller and Scott (1977) hypothesis involving Renshaw cells, the “ring” model to explain more complex locomotor patterns (e.g., backward vs. forward walking), the flexor burst generator with an assymetrical excitatory drive from a flexor burst generator as well as the unit burst generator model with symmetrically-organized burst generators (for each articulation or sets of muscles) even in absence of peripheral input. More recently, the “synergy model” from Bizzi’s group and the bipartite model (or two-level CPGs) from McCrea’s group were also proposed (Tresch et al., 1999; Bizzi et al., 2008; McCrea and Rybak, 2008).

## Conceptual organization of CPG for locomotion

### Half-center model

Sherrington used the term “half-center” to explain the spinal pathway for reciprocal inhibition. That expression was used again by Graham Brown in his model of spinal locomotor control based on his experiments showing that the basic pattern for stepping was generated entirely in the spinal cord even in absence of peripheral afferent contribution (due to narcosis) in spinal cord-transected cats, rabbits and guinea-pigs (Graham Brown, 1911, 1914). The animals, under general anesthesia, were lying on one side when stepping movements in the hindlimbs were spontaneously evoked (“narcosis progression”) after a transection of the cord at the lower thoracic level. Since the level of anesthetic used was shown to abolish proprio- and extero-ceptive reflexes but not locomotor activity, Graham Brown proposed a “half-center” model made of two groups of spinal neurons reciprocally organized and mutually inhibiting each others that were capable of producing the basic rhythm and pattern for stepping. Activity in the first group of neurons (e.g., extensor half-center) would send motor commands to motoneurons (exciting extensors), and would inhibit simultaneously the reciprocal group of neurons (flexor half-center) preventing the excitation of antagonists (silencing flexors). After a period of “depression” (e.g., fatigue, adaptation, post-inhibitory rebound) of the extensor half-center, the flexor half-drive would predominate for a new phase of activity. Despite these findings, the general opinion of scientists between 1920 and 1960 remained that basic locomotor activity largely depends upon sensory input from the peripheral nervous system. However, it is Elzbieta Jankowska and Anders Lundberg who have provided in the 1960s using intracellular recording techniques the first direct evidence supporting the existence of Graham Brown’s model (Jankowska, 2008). They identified intracellularly interneurons located in the lumbar segments of the cord (specifically in the lamina VII) that are active following FRA stimulation. Specifically, one group of neurons was found to be activated by ipsilateral FRA, a second group by contralateral FRA (coFRA), and a third group by both ipsi- and contralateral stimulation. After injection of L-DOPA and nialamide in spinal cord-transected cats, FRA stimulation evoked a high frequency burst followed by a long-lasting self-sustained series of discharges. Some neurons did not even show any short latency effects during the stimulus train. These interneurons were found to be monosynaptically excited by ventro-lateral funiculus stimulation which contains descending fibers from the reticular formation. One of the most important features was the reciprocal organization between these groups of interneurons since coFRA stimulation abolished the long-latency discharges evoked by ipsilateral FRA and vice versa. Finally, it was proposed that Ia interneurons could participate in the production of the locomotor pattern by receiving strong excitatory input from FRA interneurons given their corresponding rhythmic activity in L-DOPA-treated cats (Jankowska et al., 1967a,b). However, in the 1970s, several neuroscientists began to provide evidence suggesting that, as it is, this half-center organization can not fully explain the complex patterns of muscle activation found during terrestrial locomotion (e.g., in quadrupeds) (Grillner, 1981).

### Miller and Scott model

Sharing similarities with the half-center model, the Miller and Scott hypothesis proposed that Renshaw cells rather than fatigue are responsible for the alternation between flexion and extension (Miller and Scott, 1977). Increasing activity in one pool of motoneurons (e.g., extensors) would be gradually inhibited by a corresponding increase of recurrent inhibition. Architecturally, this model takes into account known neuronal connections—it is constituted of a closed chain of neurons to which flexor and extensor motoneurons are connected in different parts. Renshaw cells and Ia inhibitory interneurons which are part of this chain of neurons are mainly responsible for reciprocal activation of the flexor and extensor motoneurons (Bergmans et al., 1969). Simultaneously, Renshaw cells would remove reciprocal inhibition of antagonists (recurrent facilitation) via their spindle Ia monosynaptic inhibitory input onto Ia interneurons allowing the flexor excitatory drive to take over for a new phase of activity (i.e., flexion). Interestingly, by varying the tonic input to the alpha motoneurons and Ia inbitory interneurons, coactivation of the flexors and extensors may be achieved. Although, the reciprocity between flexors and extensors is nicely explained by this model, the origin of the rhythmicity itself is rather unclear (Kriellaars, 1992; Kriellaars et al., 1994). Other criticisms came from results showing that Renshaw cell activity may be inhibited during locomotor activity although discrepancies were reported during fictive locomotion whereas both classes of neurons were reported not to be essential for the production of a basic locomotor pattern in motoneurons. Other evidence against the Miller and Scott model was provided by Jordan’s group who showed that the basic locomotor pattern and Ia interneuron activity remain after i.v. injection of the nicotinic antagonist mecamylamine (MEC), which greatly reduces Renshaw cell activation (McCrea et al., 1980; reviewed in Guertin, 2009a).

### Ring model

The highly conceptual “ring” model was one of the models subsequently proposed to explain the existence of complex locomotor patterns (e.g., taking into account differences between backward or forward walking, synchronization and phase coupling between activity of the ring and the cyclic afferent input, etc.) (reviewed in Guertin, 2009a). It is made of a closed chain of at least five groups of neurons (e.g., 2 pure extensors, 2 pure flexors and 1 bifunctional) that project to the motoneurons either directly or through specific interneurons. The sequence of these projections determines the order of activation of various muscles during the step cycle. At rest, a number of ring neurons are tonically inhibited by a certain group of spinal neurons. Activation of the monoaminergic descending system would result in the inhibition of the inhibitory neurons with consequent disinhibition of the ring neurons. Activity within the ring is based on a cyclically propagated inhibitory drive that would travel at different speeds from one group to the other depending on the excitability (e.g., modulated by afferent input) of the path (“ring”) interconnecting them. The activity within the ring starts when the excitability level is raised (through disinhibition), so the neurons would discharge when not inhibited. A slow-propagated drive would activate neurons of a group for a longer period of time (e.g., pure extensor during the stance phase) whereas a fast-propagated one would activate a group of neurons for a brief moment in motoneurons to bifunctional muscles. However, this highly conceptual model has generally failed to convince most scientists in this field.

### Flexor burst generator model

During those same years, other models such as the flexor burst generator were also proposed. Pearson and Duysens introduced this model for insects and cats (Pearson and Duysens, 1976; Duysens, 1977; Duysens et al., 2013). It consists of a rhythmic excitatory drive from the flexor burst generator to populations of flexor motoneurons. The burst generator would inhibit, via an inhibitory interneuron, the activity of extensors otherwise activated during the flexor silence by a tonic excitatory input. This asymmetrical model was abandoned later on in favor of a more symmetrical bipartite organization (i.e., in which both extensor and flexor portions are equally driven). This change in views is likely related to the subsequent description of a powerful feedback system associated with ankle extensor group I afferents that can reset rhythms and strongly excite most pools of hindlimb extensor motoneurons during locomotion. This said, recent data mainly from Brownstone’s group provided evidence suggesting that an asymmetrical CPG organization should perhaps be re-considered. They proposed that a rhythm-generating layer, composed of a kernel of heterogeneous and electrotonically-coupled neurons, would project directly to the flexor half-center of the pattern formation layer (Brownstone and Wilson, 2008).

### Unit burst generator model

In the 70s and early 80s, the unit burst generator model contributed to the demonstration of a symmetrically-organized generator in the spinal cord that can produce the basic pattern of motor commands for walking even in absence of peripheral input (Edgerton et al., 1976; Grillner, 1981). It was proposed essentially to explain that locomotion is not only a strictly alternating pattern of flexor and extensor activity (requiring all motoneurons to belong to one of these two groups) as proposed by the half-center model (initially by Graham Brown and subsequently by Lundberg et al.). Patterns of locomotor activity are often complex and may include some motoneuron pools that display activity during both the flexion and extension phases of the step cycle or that display differences in the onset and offset of activity in individual flexor and extensor pools. The persistence of such complex activity patterns following bilateral deafferentation of the hindlimbs in decerebrate cats, led Grillner and Zangger (1975) to conclude that the locomotor CPG does not simply generate an alternating activation of flexors and extensors but a more complex pattern that will sequentially start and terminate disctinctively, the activity in the appropriate muscles (Grillner and Zangger, 1974). Their idea was further developed in a proposal for a CPG architecture in which separate “modules” or unit burst generators would control subsets of motoneurons. First, Szekely et al. showed that the locomotor pattern (in the forelimbs) in freely moving newts was similar before and after a bilateral section of the dorsal roots (Székely et al., 1969). This was also shown in decerebrate cats leading then to the suggestion that a CPG could exist for each joint of each limb. Activity from these “units” would be tightly coupled during “normal” walking but individually controlled by supraspinal input to produce different types of motor patterns. This model emerged after analyzing more complex patterns of locomotor activity such as backward walking, climbing, etc. For instance, it was occasionally observed during fictive locomotion that one hindlimb motor nerve can display tonic activity while the others display a normal rhythmic pattern. Also, the activity of pluriarticular muscle nerves such as *semitendinosus* is sometimes in phase with extensors, or flexors, or both, which some authors found difficult to explain with a half-center type of model. Along this idea, Bizzi’s group provided experimental and analytical results suggesting instead that sensory-dependent linear combinations of a small number of muscle synergies may generate diverse motor and locomotor patterns. Some of the cellular components of Grillner’s CPG model were identified in the 80s using a simpler non-mammalian vertebrate nervous system preparation—the *in vitro* isolated lamprey preparation (Grillner and Wallén, 1985; Grillner, 2006). However, despite the attractiveness of this proposal, the unit burst generator model has not generally explained the existence of other complex patterns of motoneuronal activity such as those found during spontaneous deletions (see section below).

### Two-level-half-center models

Thus far, most models had failed to entirely explain the many patterns that can occur in the generally alternating activity of flexors and extensors during locomotion. In particular, unpredictable changes called “deletions” which refer to periods of silenced activity in some populations of motoneurons (e.g., extensors such as the *soleus*) accompanied of sustained or rhythmic activity in antagonist motoneurons (e.g., flexors such as the *tibialis anterior*) while post-deletion rhythm is generally maintained. This is essentially why attempts to explain these other types of changes have first been proposed based on bipartite CPG levels. Indeed, a detailed analysis of nerve and muscle activity during spontaneous walking in non-paralyzed cats or during fictive locomotor activity in paralyzed decorticated cats let Perret and Cabelguen to initially propose a bipartite or two-level (half-center-like) model that explains the complex biphasic activity in so-called bifunctional motoneurons (e.g., *semitendinosus*) (Perret and Cabelguen, 1980; McCrea and Rybak, 2008; reviewed in Guertin, 2009a). They proposed that not only one half-center but both half-centers (extensor and flexor ones) would send motor commands to bifunctional motoneurons. A variety of motoneuron patterns could be produced by modulating the half-centers output “en route” to these motoneurons. They also suggested that a rhythm generator would be functionally separated from a pattern generator since the rhythm and the amplitude of the locomotor drive potentials appear to be two distinct characteristics that can be independently and spontaneously changing. This paved the way to other studies from Kriellaars and Jordan who proposed a functional separation of pattern and amplitude (Kriellaars, 1992; Kriellaars et al., 1994). They showed that locomotor drive potentials monitored simultaneously (by dual intracellular recordings *in vivo*!) in pairs of motoneurons generally covary in amplitude in homonymous motoneurons while antagonist motoneurons inversely covary. The complex locomotor pattern in bifunctional motoneurons receiving input from both half-centers would be sculpted by controlling the amplitude of the flexor and extensor locomotor drive “en route” to these motoneurons. A similar separation of CPG function (rhythm vs. pattern and amplitude) was suggested in other studies to explain how sensory stimulation can also alter locomotor cycle timing without altering the level of motoneuron activity which has served in the 90s as basis to the elaboration of the most recently proposed multi-level models (2+ and 3 levels, see Rybak’s work). Additional evidence from non-resetting deletions reported by McCrea et al. during fictive locomotion and scratch in the decerebrate cat strongly supported also this two-level CPG organization (Lafreniere-Roula and McCrea, 2005). Finally, mathematical models recently developed by McCrea and Rybak have constituted additional supports for the existence of multi-level CPG (half-center-like) organizations that can explain spontaneous deletions and other complex patterns of activity during locomotion (McCrea and Rybak, 2008; Knusel et al., 2013).

All and all, none of the above models have been refuted and yet, according to some, none of the conceptual models is capable to explain the wide variety and diversity of locomotor patterns that exist in real life.

## Cellular constituents of the CPG for locomotion

Beyond these conceptual considerations about its organization, the CPG for locomotion has been characterized in part as a group of interneurons localized mainly in the lumbar area of the spinal cord (Giszter et al., 2007; Guertin, 2009a; Guertin and Steuer, 2009). With an *in vitro* isolated spinal cord preparation from neonatal rats, Kjaerulff et al. used sulforhodamine-101, an activity-dependent marker/dye, to identify CPG neuron candidates in L1–L6 near the central canal as well as near the medial intermediate zone (Barajon et al., 1992; Kjaerulff et al., 1994). A comparable approach used by Cina and Hochman showed the existence of a restricted number of labeled cells (presumably CPG neuron candidates) more specifically in L1–L5 segmental areas (Hochman et al., 1994; Cina and Hochman, 2000). These findings are supported also by other studies that showed, using electrical stimulation or selective lesions, key rhythmogenic CPG elements specifically in L1 and L2 in mice (Nishimaru et al., 2000). This is also supported by findings from Dimitrijevic et al. (1998) in SCI patients following epidural stimulation near L1–L2 that triggered locomotor-like movements in the lower extremities (Dimitrijevic et al., 1998; Shapkova and Schomburg, 2001; Selionova et al., 2009). Observations in one patient with a complete spinal cord transectionmid-thoracically displaying spontaneous episodes of locomotor-like movements when lying in a bed could be interpreted as valuable evidence of a CPG in the lumbar cord of humans (Nadeau et al., 2010). Discrepancies may exist in other species regarding the exact localization of the CPG (e.g., in mid-lumbar segments in cats) (Langlet et al., 2005). In primitive vertebrate species such as lampreys, cellular components have been identified and even recorded from electrophysiologically nearly 30 years ago (LC cells, CC interneurons, etc.). Indeed, with its simpler neural system, it has been easier to investigate extensively neuronal activity from single spinal cells using the *in vitro* isolated spinal cord preparation from lampreys. The quest for dissecting further the CPGs has been supported also by findings obtained in parallel (or before) from non-mammalian and invertebrate species. In fact, studies on sea slugs, leeches, cockroaches, stick insects and crustacean locomotor (swimmeret) and motor (e.g., somatogastric system) pattern-generating networks have played a pivotal role in understanding further the cellular and network bases of rhythmic motor and locomotor patterns in both invertebrate and vertebrate species (Hugues and Wiersma, 1960; Pearson, 1993; Hopper and DiCaprio, 2004; Clarac and Pearlstein, 2007; Buschges et al., 2008; Harris-Warrick, 2011).

## Pharmacological and electrophysiological properties of the locomotor CPG

Since the 1980s, pharmacological manipulations using a plethora of newly available receptor ligands (agonists and antagonists) have largely contributed to understand and further describe the detailed organization of the locomotor CPG (Rossignol et al., 2001; Harris-Warrick, 2011). Experiments in *in vitro* isolated rodents (rat, mouse and turtle preparations) as well as, subsequently, in *in vivo* TX rodents (adult rat and mouse models) led to substantial advances in cellular target identification (receptors and channels) associated with locomotor rhythm generation and modulation (Guertin and Hounsgaard, 1998a,b; Guertin, 2009b). Blood brain barrier (BBB) permeable ligands have served to pharmacologically “dissect” *in vivo* the contribution of specific receptors and channels to locomotor rhythm-generation (Guertin, 2008). Clear CPG-activating effects induced by specific drugs have also been found in various *in vitro* preparations from invertebrate and vertebrate species, although it is beyond the scope of this review to report on all of them (Grillner and Wallén, 1985; Sigvardt et al., 1985; Cazalets et al., 1990; Cowley and Schmidt, 1994; Kiehn and Kjaerulff, 1996; Schmidt et al., 1998; Schmidt and Jordan, 2000; Bonnot et al., 2002; Kiehn et al., 2008). As mentioned earlier, the noradrenergic system and specifically L-DOPA has been among the first systems to be associated with CPG activation in acutely TX cats and rabbits (Jankowska et al., 1967a,b; Viala and Buser, 1969; Grillner and Zangger, 1974). Clonidine, an alpha-2 adrenergic receptor agonist, administered during sensory stimulation (e.g., tail or sexual organ pinching) was also reported to enhance the effects of training and/or sensory stimulation (remains unclear) on locomotor rhythmogenesis in TX cats (Forssberg and Grillner, 1973; Barbeau and Rossignol, 1990, 1991; Pearson and Rossignol, 1991; Chau et al., 1998a,b; Rossignol et al., 2001). However, it has been difficult to determine site-specific actions (e.g., on motoneurons, CPG neurons or primary afferents) from results in many of these earlier studies that were not designed to specifically assess drug-induced CPG activation *per se* (i.e., given the use of additional stimuli including tail stimulation, sexual organ pinching, regular training or weight support assistance, see Lovely et al., 1986; Bélanger et al., 1996; Zhang et al., 2010). More recently, experiments conducted in our laboratory in a mouse model of paraplegia (a complete low-thoracic TX) with no assistance or additional stimuli (e.g., no training, no tail stimulation, no sexual organ pinching, and no weight-support assistance to avoid unspecific non-drug induced effects) have contributed to identify clearly a subset of transmembranal receptors involved in pharmacologically-elicited, CPG-mediated locomotor-like movements in the lower extremities (Guertin, 2008, 2009a,b). For instance, L-DOPA, serotonin (5-HT) or dopamine (DA) receptor ligands such as 8-OH-DPAT, buspirone (5-HT1A/7 agonists), quipazine (5-HT2A/2C agonist) or SKF-81297 (D1-like agonist) were found to trigger significant locomotor-like movements (i.e., rhythmic bilaterally alternating flexions and extensions involving one or several hindlimb joints) whereas others ligands such as 3-Trifluoromethylphenylpiperazine (TFMPP) (5-HT1B), m-CPP (5-HT2B/2C), SR57227A (5-HT3) or clonidine (adrenergic alpha-2 agonist) were shown to elicit mainly non-locomotor movements (i.e., non-bilaterally alternating movements, twitches, cramps, etc.) in TX mice (Guertin, 2004, 2005; Landry and Guertin, 2004; Guertin and Steuer, 2005; in rats, see Antri et al., 2005; Lapointe et al., 2008; Ung et al., 2008). Using selective antagonists and genetically-manipulated animals (e.g., 5-HT7KO mice), it has been clearly established that N-Methyl-D-aspartate (NMDA), 5-HT1, 5-HT7, 5-HT2A and D1 receptors were specifically involved in mediating such CPG-activating locomotor-like effects (Landry et al., 2006a; Lapointe et al., 2009). For instance, endogenous glutamate release and NMDA receptor activation were reported as critically important for quipazine-induced effects since a complete loss of induced movement was found in NMDA antagonist (MK-801)-treated animals previously pretreated with NMDA (Guertin, 2004). Regarding DA receptors, administration of D2, D3 or D4 agonists was found not to generate significant hindlimb locomotor-like movements whereas D1/D5 agonists such as SKF-81297 can potently elicit locomotor-like movements that are lost in selective D1-like (D1/D5) antagonist-pretreated TX mice but no in D5 –/– paraplegic TX mice suggesting a specific contribution of the D1 subtype to CPG activation (Lapointe et al., 2009). All and all, pharmacological approaches in *in vivo*, untrained and non-assisted TX animals have contributed to identify a subset of CPG-activating compounds (and corresponding receptors confirmed with selective antagonists and knockout animal models) (Guertin, 2009a, 2012). However, none of these molecules were found to generate large amplitude weight bearing stepping movements *per se* in untrained, non-assisted and non-sensory stimulated TX animals suggesting that only partial CPG activating effects can be achieved using these ligands administered separately. Subsequent studies conducted in our laboratory have shown that only simultaneous activation of some of these candidate receptors can, using similar pharmacological approaches and animal models, induce full locomotor-inducing effects. Partial CPG-activating effects (i.e., associated with crawling rather than full weight bearing stepping) induced by some ligands, as mentioned above, were found indeed to turn into full CPG-activating effects (i.e., weight bearing stepping in non-assisted, untrained and non-stimulated paraplegic animals) by simultaneously administrating 8-OH-DPAT, quipazine, L-DOPA, or SKF-81297 (Guertin, 2009b; Guertin et al., 2010, 2011). Prior to these findings, numerous pharmacological studies aimed at improving locomotor function recovery after SCI have been also conducted in other *in vivo* models of SCI (e.g., in TX cats). However, it has remained difficult to consider those results as evidence of cellular targets associated with CPG activation or modulation given the used paradigm (experiments conducted during tail stimulation, sex organ pinching, body-weight supported, movement-assisted manually, etc.) (Forssberg and Grillner, 1973; Lovely et al., 1986; Barbeau and Rossignol, 1991; Bélanger et al., 1996). Some of our findings, reproduced subsequently in other laboratories, have thus been independently validated (Courtine et al., 2011; van den Brand et al., 2012).

This said, the extent to which the cellular network activated pharmacologically *in vivo* corresponds to already identified CPG neuron candidates (electrophysiologically or genetically) remains unclear (see section below). However, dual immunohistochemical experiments recently showed locomotor activity-labeled (c-fos) 5-HT1A-, 5-HT2A- or 5-HT7- positive neurons in the cat lumbar cord (specifically in laminae VII-VIII, Noga et al., 2009) suggesting that some of the locomotor activity-related receptors identified recently in *in vivo* models may indeed be located on CPG neurons (Hochman et al., 2012).

## Specific ionic conductances and channels

This is also an aspect of the CPG that remains incompletely understood. It is generally accepted that one of the main cellular features expected to be found in CPG neuron candidates is its capacity to express endogenously rhythmic activity during fictive or real locomotion (Eken et al., 1989; MacLean et al., 1997; Brownstone and Wilson, 2008; Brocard et al., 2010). For several decades, it was also generally accepted that some neurons of the CPG were expected to express specifically autorhythmic or pacemaker-like properties given that locomotion is, by definition, a rhythmic motor behavior. There have been indeed several cell populations in the corresponding area of the CPG (i.e., lumbar segments in mammalian species) shown in the last few decades to be capable of expressing pacemaker-like properties in specific conditions such as in the presence of NMDA alone or combined, at lower dose, with serotonin and DA (e.g., bath-applied in the case of *in vitro* isolated spinal cord preparations) (Wang et al., 2006; Grob and Guertin, 2007). Plateau potential is another intrinsic property believed by some researchers to be associated with endogenous (tetrodotoxin-resistant) pacemaker-like activity (Eken et al., 1989). Transmembranal currents and channels underlying both of these properties (voltage oscillations, NMDA ionophore, L-type (CaV1.3) Ca^2+^ channel, persistent Na^2+^ current, I_h_ current, I_A_ current, I_CAN_, I_NaP_ current, I_K(Ca)_ current, post-inhibitory rebound have been relatively well-characterized in lamprey, turtle, tadpole, rodent and cat preparations with some species-dependent specificities (Grillner and Wallén, 1985; Reith and Sillar, 1998; Harris-Warrick, 2002, 2011; Grillner, 2006; Wang et al., 2006; Grob and Guertin, 2007). Nonetheless, the specific contribution of these properties (in motoneurons and some interneurons) to real life locomotion remains speculative and a source of debate.

## Genetically-identified neurons of the CPG for locomotion

In recent years, advances in genetics and transgenic murine models have largely contributed to the identification of specific CPG neuron candidates *per se* (Ginty et al., 1992; Lanuza et al., 2004; Goulding, 2009). Targets initially identified in the developing neural tube (i.e., 11 distinct populations of spinal neurons (dI1–dI6, V0–V3, VMN) based on the expression of transcription factors (e.g., Jessell’s work) have largely contributed to these findings. Several populations of interneurons involved in locomotor activity were indeed characterized genetically (e.g., V0–V3). One of them is the population of V0 interneurons that was associated with left-right alternation since mice without V0 interneurons (lacking the transcriptional factor Dbx1) displayed bilateral synchrony rather than bilateral alternation during locomotion. Another population referred to as the V1 inhibitory interneurons (expressing the transcription factor Engrailed 1) was associated with high locomotor frequencies since slow rhythms were found in En1-DTA mice (Gosgnach et al., 2006). Other genetically-identified populations include the Chx10-expressing cells (V2a glutamatergic and V2b gabaergic interneurons) located in the intermediate zone of the gray matter in the lumbar spinal cord (Lundfald et al., 2007; Crone et al., 2008). These neurons were associated with frequency, amplitude and bilateral coordination since all of these parameters were affected in Chx10-DTA mice (lacking V2a interneurons). V3 interneurons (Sim-1 expressing cells) constitute another recently identified population shown to participate in the production of a robust and balanced rhythm during locomotion. Indeed, rhythmic activity was found to be partially disrupted in mice lacking V3 interneurons (Zhang et al., 2008). Genetically-engineered animals were also utilized to show that hopping instead of normal walking is displayed in mice lacking the ephA4 receptor-expressing lumbar interneurons. Finally, another population of CPG neuron candidate called HB9 neurons was reported to provide neuronal excitation during locomotion. Although, no corresponding knockout model has been tested, compelling evidence suggests that the HB9 excitatory interneuron belongs to an asymmetrically-organized rhythm-generating network (Brownstone’s and Jessell’s work). Taken altogether, results from these exciting new studies in mice suggest that these genetically-characterized interneurons (V0–V3, EphA4, HB9) may constitute different cellular components of the CPG (Kullander et al., 2003; Wilson et al., 2005; Brownstone and Wilson, 2008). For instance, V0 interneurons form many cell types including commissural neurons which could establish inhibitory reciprocal connections between the two sides of the spinal cord. In turn, V1 interneurons may be associated with inhibitory interneurons such as the Ia inhibitory and Renshaw cells whereas HB9s may be excitatory interneurons constituting at least part of the rhythm generating layer. However, additional studies need to be conducted in order to fully characterize these promising new CPG neuron candidates (Butt et al., 2002; Wilson et al., 2007). It is important to mention also in most cases of gene deletions using these KO models, elimination of rhythmic motoneuron excitation or rhythmic inhibition is rarely found suggesting that several CPG secrets remain to be described.

## Characteristics of other CPGs

### Spinal generator for ejaculation

A breakthrough finding in 2002 has drastically changed our view of the neurobiology of sexual function (Truitt and Coolen, 2002; Coolen et al., 2003). Indeed, Truitt and Coolen have shown that ejaculation critically depends upon a spinal generator or network of neurons referred to as the Spinal Generator for Ejaculation (SGE; Truitt and Coolen, 2002). Located in the lumbar segments L3 and L4, the SGE is defined as a circuit capable of producing self-sustained rhythmic output to pudendal motoneurons. The SGE was found to contain a key population of neurons (lumbar spino-thalamic neurons also called LSt cells) that (1) project to forebrain; (2) project to pudendal motoneurons (correspond to those located in the Onuf’s nucleus in men); and (3) receive input from sexual organs via the pudendal and dorsal nerve of the penis. In fact, ejaculation is completely lost in animals undergoing LSt cell-lesioned procedures (using SSP-saporin). LSt cells are found in the vicinity of the central canal (lamina X and medial portion of lamina VII, and most of them specifically contain galanin, CCK, enkephalin and NK-1 receptors. Electrical stimulation of the pudendal or dorsal nerve of the penis nerves was shown to elicit ejaculatory responses in low-thoracic Tx rats. Comparable data were found in SCI men supporting the existence of LSt cells (or at least of a SGE) in humans as in other species. However, the type(s) of neurotransmitters involved and the subset of post-synaptic receptors associated with SGE activation and ejaculation remain largely unknown (Courtois et al., 2008, 2013).

This said, only a few families of compounds are known to modulate sexual function (either erection, ejaculation or both) (McKenna et al., 1991; Pomerantz et al., 1993; Vargas et al., 2004; Moreland and Makela, 2005; García-Bravo et al., 2006). For instance, Guttmann and Walsh showed (earlier case reports also exist) that the cholinesterase inhibitor prostigmine administered intrathecally (i.t.) could elicit spontaneous erection accompanied sometimes with ejaculation in SCI men (Guttman and Walsh, 1971). Midodrine (alpha-1 agonist) was also shown to induce either normal (anterograde—i.e., outside the urethral meatus) or abnormal (retrograde—i.e., back inside into the bladder) ejaculation in patients with SCI but, as with cholinesterase inhibitors, it was also found to increase blood pressure and autonomic dysreflexia (Jonas et al., 1979; Riley and Riley, 1982; Staerman et al., 2001; Blanchard-Dauphin et al., 2005; Courtois et al., 2008). In animal models, muscarine (i.p. or i.t.) was found to promote erection and sometimes ejaculation in sensory-stimulated Tx rats (i.e., penile sheath retraction known as the “penile reflex” model) (Durán et al., 2000). Alpha-1 agonists such as methoxamine (which, unlike midodrine, crosses the BBB) were reported to promote reflexively-induced ejaculation (either immediately after Tx or by urethral stimulation with fluid injection) in freely moving Tx rats. P-chloroamphetamine (amphetamine derivative that increases 5-HT release) was shown to elicit fictive ejaculation (Electromyogram (EMG) correlates) in anesthetized Tx rats) (Stafford et al., 2006). Supporting a role for subclasses of spinal 5-HT receptors, 5-HT receptor agonists such as m-CPP (5-HT2) or 8-OH-DPAT (5-HT1A) were also found to promote sometimes ejaculation in sensory-stimulated Tx (self-grooming) or non-Tx (copulating) rats (Camacho et al., 2007). However, mixed 5-HT-induced effects have also bee reported suggesting that serotonergic modulation may depend upon the site of action and route of delivery (inhibitory effects in brain vs. excitatory effects in spinal cord) (Yonezawa et al., 2008). Regarding DA agonists, none have been tested in Tx animals although D2-like agonists i.c.v. injected was reported to promote ejaculation in intact rats.

### Spinal micturition center

Micturition essentially depends, for the storage and periodic elimination of urine, on the coordinated activity of smooth and striated muscles in the several functional units of the lower urinary tract, namely the urinary bladder, the bladder neck, the urethra and the urethral sphincter (Mallory et al., 1991; de Groat et al., 1993; Andersson and Pehrson, 2003; Sugaya et al., 2005). The coordination between these organs is mediated by a complex neural control system that is located partly in the spinal cord (Schrøder, 1985; Birder and de Groat, 1993; Birder et al., 1999; Dolber et al., 2007). Central interneurons retrogradely labeled by injection of pseudorabies virus into the urinary bladder of the rat were found in regions receiving afferent input from the bladder (Nadelhaft and Vera, 1995; Sugaya et al., 1997). A comparable distribution was shown following injections of virus into the urethra (Vizzard’s work) or the external urethral sphincter (EUS), indicating a prominent overlap of the interneuronal pathways controlling the various target organs of the lower urinary tract (Vizzard et al., 1995). In addition, spinal interneurons (located near dorsal commissure, superficial dorsal horn and sacral parasympathic nucleus) involved in processing afferent input from the lower urinary tract have been identified by c-fos expression following noxious or non-noxious stimulation of the bladder and urethra in rats (de Groat’s work). Some of these interneurons send long projections to the brain, whereas others make local connections in the spinal cord and participate in segmental spinal reflexes. These results provide strong evidence of a CPG for EUS control both in the thoracolumbar (T8-9 for storage of urine) and lumbosacral (L3-L4, L6-S1 for expulsion of urine) spinal cord. Various neurotransmitters have been associated with the control of the lower urinary tract including glutamate, tachykinins, pituitary-adenylate-cyclase-activating polypeptide, NO and ATP. Glutamate, acting on NMDA and non-NMDA receptors, seems to be critically involved in spinal and supraspinal reflex pathways that control the bladder and the EUS (Yoshiyama and de Groat, 2005). In contrast, GABA (γ-aminobutyric acid), glycine and enkephalins were reported to exert a tonic inhibitory control in the pontine micturition center (PMC) and regulate bladder capacity. Other neurotransmitters including DA, 5-HT, NA, and acetylcholine have either inhibitory or excitatory effects, depending on the type and location of activated receptors (de Groat et al., 1993; de Groat and Yoshimura, 2001; Chang et al., 2006; Dolber et al., 2007). For example, DA elicits inhibitory effects on micturition through D_1_-like receptors and facilitatory effects through D_2_-like receptors. Also, much like what has been shown with 5-HT1A/7 receptor agonists on CPG activation (Landry et al., 2006a), 5-HT1A/7 receptor agonist 8-OH-DPAT was found to induce rhythmic EUS relaxation during voiding in urethane-anesthetized chronic Tx rats. Other potential pharmacological treatments to facilitate micturition for instance after SCI; are (1) intravesical administration of drugs such as vanilloids which is aimed at desensitizing bladder afferents (Fowler et al., 1994); (2) injection of BoNT/A into the detrusor to temporarily block the pre-synaptic release of ACh from the parasympathetic innervation and produce a paralysis of the detrusor smooth muscle (Schurch et al., 2000); and (3) administration of NO donors such as isosorbide dinitrate to produce a significant reduction in striated sphincter pressure at rest and during dyssynergic contraction (Reitz et al., 2004).

### Spinal defecation center

Bowel control involves a complex interactions between the autonomic (sympathetic and parasympathetic), central (brain and spinal cord) and muscular systems (sphincters and smooth muscles) (Rostad, 1973). Experiments in spinal cord-transected rats (cervical level) have clearly shown the existence of a reflex defecation center (also known as the lumbosacral defecation center) located by retrograde labeling at the low lumbar and upper sacral level (i.e., L6-S1 in rats) (Shimizu et al., 2006). Evidence of increased defecation in humans caused by Lumbosacral Defecation Center (LDC)-mediated effects, induced by ghrelin receptor agonists, has been shown whereas direct evidence using intrathecal injections have been reported in animal models (Shimizu et al., 2006). Indeed, defecation induced pharmacologically was prevented by cutting the nerve pathways that connect the lumbosacral spinal cord and the colorectum, but not by severing the thoracic spinal cord (Ferens et al., 2011; Pustovit et al., 2014). Unfortunately, Ghrelin or its known agonists (e.g., ulimorelin) do not elicit defecation when administered orally and may increase blood pressure when delivered i.t.

## CPGs in humans: Evidence from direct stimulation and spontaneous plasticity and role of feedback and feedforward

Some people are reluctant to believe that spinal pattern generators or CPGs exist in humans given the lack of direct evidence. It is true that most data have been obtained from animal models, as described in this review. Experiments and tools used to explore CPG properties in animals are simply too invasive for comparable exploration in humans. But again, given that the spinal cord, among all structures of the CNS, is particularly well-preserved phylogenetically, it may be more reasonable to argue the opposite—are there evidence that humans do not have spinal CPGs given that all vertebrate species examined thus far stongly suggest the existence of comparable neural control systems for locomotion and other stereotyped motor behaviors?

The answer is “no”, we do not have evidence against the existence of spinal CPGs in humans (Bussel et al., 1989, 1992). There is increasing evidence suggesting the existence of comparable spinal networks in primates including humans—at least for locomotion (Tan, 2006a,b, 2008). For instance, epidural or intraspinal stimulation near L1–L2 to successfully trigger locomotor-like movements in the lower extremities of completely SCI patients (Dimitrijevic et al., 1998; Holinski et al., 2011; Moshonkina et al., 2012). Given that electrical stimulation of CPG does not generally lead to full weght bearing stepping (e.g., often only to air-stepping or locomotor-like movements lying in bed, this suggests that only partial CPG activation may be achieved electrically. In turn, it has been possible to activate the locomotor CPG by administration i.t., of a variety of pharmacological agents to acutely spinalized marmoset monkeys (Callithrix jacchus) in the absence of phasic afferent input to the spinal cord (Fedirchuk et al., 1998). Spontaneous expression, within weeks or months post-SCI, of rhythmic movements (myoclonus or locomotor-like movements) in humans have also been reported (Pozos and Iaizzo, 1991; Bussel et al., 1992, 1988; Calancie et al., 1994; Chervin et al., 2003; Calancie, 2006; Consentino et al., 2006; Gerasimenko et al., 2008, 2010; Nadeau et al., 2010; Field-Fote et al., 2012; Gorodnichev et al., 2012; Rye and Trotti, 2012).

How could such spontaneous movements occur (Clemens et al., 2006; Harkema, 2008; Nadeau et al., 2010)? No clear answer exists in humans (Calancie et al., 1994; Byrnes et al., 2006). However, related spontaneous plasticity events have clearly been shown in animal models to be associated with some immediate early gene expression (IEG) in CPG-corresponding areas of the spinal cord. Changes in IEG expression levels post-SCI were associated also with a cascade of subsequent changes (TNF-α, NOS; C1qb, Galectin-3, and p22(phox), glycine receptors, GAD-67, 5-HT1A/7 receptors; NGF, BDNF, and NT-3) and spontaneous spinal locomotor-like activity (Chi et al., 1993; Yakovlev and Faden, 1994; Giroux et al., 1999; Tillakaratne et al., 2002; Gómez-Pinilla et al., 2004; Landry et al., 2006b; Lukacova et al., 2006; Peng et al., 2006; Schmitt et al., 2006; Giszter et al., 2007; Li et al., 2007; Ung et al., 2007).

It is clear though that despite direct stimulation electrically or pharmacologically, spinal-mediated stereotyped motor functions such as locomotion requires at least some feedforward and feedback mechanisms for goal-oriented functional recovery. Interestingly, some feedback mechanisms may remain operational despite severe spinal cord lesions at the thoracic or cervical level. Indeed, it has clearly been shown in Tx cats, displaying some restored spinal-mediated locomotor activity on a motorized treadmill, that adaptation to different speeds remains possible (Edgerton’s or Rossignol’s work). However, to reach more complex levels of adaptation such as in climbing steps, feedforward mechanisms are probably required. Recent experiments in partially Tx rats showed that at least some descending fibers need to be intact or functional for animals, driven to walk by CPG-activating drug cocktails (such as those identified in Guertin’s laboratory), for displaying complex patterns such as walking up stairs and changing directions during walking (SŁawinska et al., 2012; van den Brand et al., 2012). It is unclear which descending systems are most important for such key feedforward mechanisms, however, cells from the parietal cortex and cerebellar systems are key to visually-guided locomotion whereas brainstem circuits and corresponding descending tracts (e.g., vestibulo-spinal tracts) are known for significant roles in equilibrium and tonus (Gurfinkel and Shik, 1973; Aydogdu et al., 2011).

All in all, future drug treatments identified in animal models to modulate spinal network activity are thus likely to be associated with comparable efficacy data in humans given significant similarities in spinal cord structures and cellular properties from lampreys to primates. However, the role of feedback and feedforward mechanisms in patients with SCI that will be given CPG-modulating and reactivating-drug treatments remains to be explored.

## Further evidence from pharmacological reactivation of the corresponding spinal networks

As mentioned above and in different sections throughout this review, it is clear that different types of CPGs exist and a wide variety of responses may be triggered pharmacologically pending upon the classes and subclasses of transmembranal receptors activated or blocked by specific ligands or exogenous neurotransmitter administration (Johnston et al., 2003; Guertin and Steuer, 2009).

In my laboratory, an extended series of drug screening experiments has been conducted in recent years. Using an experimental model of complete SCI (a low-thoracic spinal cord transected mouse) that eases and accelerates the identification of CPG-activating compounds (i.e., given the completeness and high reproducibility of this exact type of injury), we identified families of ligands capable of inducing, within minutes post-administration (s.c., p.o. or i.p.), either episodes of locomotor movements in the hindlimbs, micturition, defecation or ejaculation. Given that spinal networks controlling these functions are located below injury level in our model and that brain-mediated effects through descending inputs are unlikely in Tx mice, then such well-coordinated and stereotyped motor behaviors must be induced, at least partly, by corresponding spinal network reactivation following administration of specific drug(s).

Reactivation of spinal catecholaminergic receptors using L-DOPA/carbidopa or DA receptor agonists and 5-HT1A receptors using buspirone or 8-OH-DPAT was found to trigger 45-min episodes of basic straightforward walking in chronic Tx mice (see Guertin, 2004 work since 2004). This patent-protected technologically, now referred to as Spinalon™, was shown to elicit comparable effects in other species (patent application WO 2006026850) whereas repeated administration was found to prevent muscular atrophy and anemia among other biological functions and systems. L-DOPA/carbidopa and buspirone were shown not to have drug-drug interactions *in vitro* and *in vivo*, providing evidence of safety in animals (Guertin, 2009b; Guertin et al., 2010, 2011). Clinical trials (Phase I/IIa) in paraplegic and tetraplegic patients that began recently (December 2013) may provide further evidence of safety in patients while possibly reporting the first evidence ever of efficacy in humans for a CPG-activating drug therapy.

Given that no sensory stimulation, no harness and no electrical stimulation are used for these tests in motor-complete SCI patients, if efficacy data in ASIA-As are to be found, this could be considered as clear evidence of a CPG in humans that remains functional and reactivable with drugs despite a SCI at higher (rostral) levels. Upon positive results, a Phase IIb and eventually a pivotal Phase III shall be undertaken to eventually meet criteria for new drug approval (NDA) in the U.S., Europe and Canada expected for the end of 2018.

Given the successes obtained with this approach, additional drug screening studies were conducted in our laboratory to identify also Sacral or Spinal Micturition Center (SMC), LDC and SGE activating compounds (Guertin, 2005, 2008, 2009b). We recently identified leads and provided proof-of-concept data *in vivo* clearly demonstrating that pharmacological reactivation of these other networks upon systemic drug delivery is possible at least in animal models of complete SCI.

A combination of transmembranal receptor agonists (that can not yet be revealed for intellectual property reasons) found to elicit superior effects was selected as our final candidate product suitable for full preclinical and clinical development. Now referred to as Micselon™, this drug combination was shown to elicit, within 15 to 30 min, episodes of micturition in SCI mice suffering of chronic urinary retention (patent application US 61/946,097). Again, the well-coordinated motor responses triggered by this therapy in completely SCI animals that induce in absence of sensory stimulation, providing strong evidence of SMC-activating effects. Lack of drug-drug interactions and evidence of efficacy in SCI patients remain to be shown prior to IND and NDA approval of this new experimental therapy against urinary retention.

A comparable technology was also found to restore some defecatory activities in SCI animals. Leads were identified using our technological platform adapted to assess defecation in paraplegic mice. Proof-of-concept data *in vivo* were obtained recently and Optimization phase studies with drug combinations revealed superior defecation-eliciting effects with a bi-therapy referred to as Transilon™. It was found to elicit within 30 min episodes of defecation in chronic SCI mice suffering of constipation (patent application US 61/946,113). Again, the well-coordinated motor responses triggered by this therapy in animals in absence of sensory stimulation provide strong evidence of LDC-activating effects. Lack of drug-drug interactions and evidence of efficacy in SCI patients remain to be shown prior to regulatory approval of this new experimental therapy against chronic constipation (expected by 2020).

Finally, we found a 4th class of drug treatments capable of spinal network reactivation in completely SCI subjects. Lead compounds were identified although Optimization phase studies have not been completed yet. Nonetheless, we found compounds and specifically drug combinations (which specific identity can not be revealed yet for intellectual property reasons) that can elicit unconditionally (with or without erection) seminal emission alone or seminal emission accompanied of seminal expulsion (full ejaculatory motor pattern) in male Tx mice (patent application US 60/996,535). These results provided proof-of-concept evidence of efficacy (SGE activation) in *in vivo* animal models of SCI. Again, additional tests remain to be performed to show comparable efficacy in humans and thus to demonstrate the existence of SGE neurons remaining functional and reactivable pharmacologically in people with SCI suffering of anejaculation.

In conclusion, CPGs involved in the control of several stereotyped motor behaviors such as locomotion, micturition, defecation and ejaculation have clearly been shown in a wide variety of species (Vizzard et al., 1995; Dimitrijevic et al., 1998; Truitt and Coolen, 2002; Shimizu et al., 2006; Clarac and Pearlstein, 2007; Guertin, 2009a; Guertin and Steuer, 2009). The one underlying pattern and rhythm generation for locomotion is by the far the most extensively studied although cellular characterization remains incomplete. Other CPGs, although poorly characterized cellularly, have also been clearly shown in different species. Nonetheless, this has not prevented research from identifying drug treatments capable of reactivating these networks separately after SCI at higher (rostral) levels. In fact, several experimental drugs (drug combinations composed of known and rather safe molecules) are currently in development clinically or preclinically. These new pharmacological aids may provide both, further evidence of spinal CPGs in humans and, useful noninvasive approaches to temporarily restore some key biological functions in people with SCI or comparable neurological disorders (e.g., multiple sclerosis) (Holmes, 1915; Fung et al., 1990; Wainberg et al., 1990; Rémy-Néris et al., 1999; Edgerton et al., 2001; Guertin and Steuer, 2009; Thorpe et al., 2011; Dietz and Sinkjaer, 2012; Guertin, 2012; Harkema et al., 2012; Jones and Cavanna, 2012; Schürks and Bussfeld, 2013).

## Conflict of interest statement

Pierre A. Guertin is professor in neurosciences at Laval University as well as CEO of a biopharmaceutical company, Nordic Life Science Pipeline Inc.

## Abbreviations

CPG: Central Pattern Generator
SMC: Sacral or Spinal Micturition Center
LDC: Lumbosacral Defecation Center
SGE: Spinal Generator for Ejaculation
TX: Spinal Cord Transection
SCI: Spinal Cord Injury
NMDA: N-Methyl-D-aspartate
5-HT: Serotonin
DA: Dopamine
CNS: Central Nervous System
EMG: Electromyogram.
